# Best medical practices for older adults after a disaster: a narrative review

**DOI:** 10.3389/fmed.2024.1420495

**Published:** 2024-10-25

**Authors:** Aiko Ishiki, Emiko Kurosawa, Daiki Narai, Yuta Sakai, Youtaro Arima, Akinobu Aihara, Toshihiro Yamagata, Yoshihisa Katsuta, Shigeto Mashiko, Tomoya Oizumi, Katsutoshi Furukawa

**Affiliations:** Division of Geriatric and Community Medicine, Faculty of Medicine, Tohoku Medical and Pharmaceutical University, Sendai, Japan

**Keywords:** disaster, earthquake, tsunami, evacuation, older people

## Introduction

Traditionally, New Year's Day is a joyous occasion for family gatherings in Japan. Many young workers and students usually return to their rural homes from urban districts where they work or study during the New Year break. On Monday, 1 January 2024, at ~04:10 pm, an earthquake with a magnitude of 7.6, followed by a tsunami, struck the Noto Peninsula in Japan (1). More than 200 casualties have been reported to date. While the percentage of individuals over the age of 65 is 9.3% globally and 28.6% in Japan, Ishikawa Prefecture, where the Noto Peninsula is located, has an even higher aging rate of 30.1%, with Suzu City reaching 51.4%.

Among the evacuees at the Ishikawa Sports Center in Kanazawa, 80% were elderly and 60% required nursing care. This marks the second time that Japan's super-aged society has been impacted by a major earthquake and tsunami, the first being the Great East Japan Earthquake on 11 March 2011 (2). This narrative review explores the impact of this earthquake, as well as the preparedness and response efforts in relation to older people.

## Disaster responses

### Acute responses

Immediately after the earthquake and tsunami occurred, several disaster-relief teams, such as the Disaster Medical Assistance Team (DMAT), responded to the Noto Peninsula to rescue victims and provide emergency medical care. It is critical that the lives saved during the disaster are not lost later due to environmental challenges, as disaster-related secondary deaths should be prevented. Older people accounted for the majority of disaster-related deaths (3).

### Health problems of the elderly

Older people often suffer from chronic illnesses that require regular medication (4). Their weakened immune systems also put them at high risk of infection. Additionally, during disasters, they are vulnerable to conditions such as stroke, heart failure, thrombosis, or infectious diseases due to limited access to clean water, nutritious food, and shelter. Poor hygiene and cold conditions in evacuation facilities further exacerbate these risks (5). Research confirms that older people are at a significantly higher risk of developing pneumonia in such situations (6).

### Prevention of health problems

To prevent the worsening of chronic diseases, hypothermia, infectious diseases, and other health issues, it is necessary to ensure that older individuals receive the following: appropriate medication, hydration, nutritious food, the opportunity to exercise, oral care, proper hygiene, and warm shelter (7). In some facilities, patients with respiratory infections, such as influenza and COVID-19, and gastrointestinal infections, such as norovirus, have also been reported (8). Additionally, preventive measures are needed for people sheltering outside of evacuation centers, such as those staying crumbling homes or cars. Overcrowding in shelters makes it difficult to practice preventive measures such as hand washing and gargling (9). As physical strength and immunity decline due to cold sleeping conditions, it is essential to move residents to secondary evacuation facilities, such as hotels, public housing, or rented accommodations, as early as possible (10).

### Relocation stress

Many people may be reluctant to move to secondary evacuation sites due to the emotional stress of leaving behind their homes and belongings. This reluctance can lead to decreased physical strength, motivation, cognitive function, and the inability to perform daily activities, contributing to “relocation stress.” This stress is often marked by increased falls, higher susceptibility to pneumonia and infections, and mental health issues that can result in disaster-related deaths. It is crucial to provide adequate support and collaborate with residents to develop favorable plans for relocation (11).

## Impact on older adults

Quantitatively, older adults are significantly more at risk of mortality in the aftermath of disasters. For example, during Hurricane Katrina, older adults accounted for ~70% of fatalities, despite comprising only a small percentage of the population (12). Similarly, following the 2011 tsunami in Japan, older survivors experienced higher rates of hypothermia and dehydration (2). Qualitatively, older adults are more likely to be displaced from their homes and face challenges in accessing medical care. Research has shown that after the 2010 Haiti earthquake, older adults faced significant barriers to accessing healthcare services, with only a fraction of them receiving necessary follow-up care (13).

## Discussion

For older people, changes in their living environment and relationships with those around them can negatively impact both their physical and mental wellbeing, even if they were previously healthy (14–16). To prevent relocation stress, responders must be mindful of these risks and encourage family members and community members to interact and engage with older people. Keeping older people active by encouraging them to move, exercise, and resume their daily activities is also beneficial. A consistent, proactive approach is essential, such as ensuring appropriate medical care and encouraging prompt consultation with healthcare professionals if any changes in their physical condition arise (17, 18).

Understanding “relocation stress” represents a significant gap in disaster response efforts, as some responders, including medical staff, may not fully grasp its importance or the risks it poses. We believe that two key areas of research are vital moving forward: (i) conducting thorough and careful health check-ups of older adults, and (ii) investigating which interventions most effectively improve the health of elderly people during disaster recovery. Our conclusions are supported by the Japan Geriatrics Society.

There is a pressing need to focus on the overall wellbeing of disaster-affected individuals, addressing both their physical and mental health while providing long-term support for their recovery and reconstruction efforts.
